# Intracellular Signaling Pathways and Their Potential Targeting for Treatment of Ocular Posterior Segment Fibrosis

**DOI:** 10.18502/jovr.v20.16966

**Published:** 2025-05-21

**Authors:** Tahmineh Motevasseli, Aryan Seraj, Narsis Daftarian, Mozhgan Rezaei Kanav, Hamid Ahmadieh, Nader Sheibani

**Affiliations:** ^1^Ophthalmic Research Center, Research Institute for Ophthalmology and Vision Science, Shahid Beheshti University of Medical Sciences, Tehran, Iran; ^2^Experimental Medicine, Department of Medicine, The University of British Columbia Faculty of Medicine, Vancouver, British Columbia, Canada; ^3^Arthritis Research Canada, British Columbia, Canada; ^4^Ocular Tissue Engineering Research Center, Research Institute for Ophthalmology and Vision Science, Shahid Beheshti University of Medical Sciences, Tehran, Iran; ^5^Department of Ophthalmology and Visual Sciences, University of Wisconsin School of Medicine and Public Health, Madison, WI, USA; ^6^McPherson Eye Research Institute, University of Wisconsin School of Medicine and Public Health, Madison, WI, USA; ^7^Department of Cell and Regenerative Biology, University of Wisconsin School of Medicine and Public Health, Madison, WI, USA

**Keywords:** Age-related Macular Degeneration, Diabetic Retinopathy, Ocular Fibrosis, Proliferative Vitreoretinopathy, Tgfβ Signaling

## Abstract

Treatment of posterior segment fibrosis is an unmet challenge in ophthalmology. Fibrotic responses complicate the pathology and treatment of age-related macular degeneration, diabetic retinopathy, retinal detachment, and other retinal diseases resulting in severe visual impairment. There is a lack of clear understanding of the exact mechanisms and different cell types taking part in retinal and preretinal fibrosis. This review discusses the current knowledge regarding various aspects of the intracellular signaling pathways impacting vitreoretinal fibrotic processes, focusing on the cellular and molecular mechanisms, summarizing the results of preclinical and clinical studies, and suggesting strategies for future investigations.

##  INTRODUCTION

Following inflammation and metabolic dysfunctions in the damaged posterior eye segment, the normal hemostatic processes are dysregulated, and a healing reparative response is activated. The dysregulated chronic responses lead to increased vascular permeability, inflammatory cell infiltration, myofibroblast cell proliferation and activation, extracellular matrix (ECM) accumulation, and ECM remodeling and tissue contraction. Fibrosis is the result of excessive proliferation and deposition of ECM components by fibroblasts, myofibroblasts, and inflammatory cells.^[1]^ Epithelium–mesenchymal transition (EMT) and activated cells converting to myofibroblasts are the principal processes involved. However, when these processes become chronic, they cause severe damage to ocular structures with a profound impact on vision.^[2]^ Mechanical injury, inflammatory, ischemic, or degenerative diseases promote tissue fibrosis. Factors such as transforming growth factor- β (TGF β ), connective tissue growth factor (CTGF), vascular endothelial growth factor (VEGF), platelet-derived growth factor (PDGF), tumor necrosis factor- α (TNF α ), and pigment epithelium-derived growth factors with profibrotic activity play important roles in the posterior segment fibrosis.^[3]^

Preretinal fibrosis is a complex biological process responsible for inducing retinal pathology and therapeutic failure. In diabetic retinopathy (DR), ischemia induces retinal neovascularization, which can lead to ﬁbrovascular proliferative tissue formation and tractional retinal detachment. Subretinal ﬁbrosis is the most common result of macular neovascularization secondary to neovascular age-related macular degeneration (nAMD) and pachychoroid neovasculopathy (PNV). Subretinal fibrosis mostly develops during the natural course of nAMD. It can also occur and even be enhanced following treatment with intravitreal injections of anti-VEGF drugs. This causes severe visual impairment as the neovascular membrane transforms into a fibrovascular structure and scar formation, leading to local destruction of photoreceptors and RPE cells.^[4,5]^ Additionally, retinal detachment or previous vitreoretinal surgery can induce fibrosis and progress to proliferative vitreoretinopathy (PVR), which is the most common cause of failure in retinal detachment surgery.^[5,6]^

Excessive deposition of ECM proteins, EMT, and appearance of myoﬁbroblasts and inﬂammatory cells are major events reported in fibrosis in all retinal disease cases.^[6,7]^ In the present review, we summarize the mechanisms of posterior segment fibrosis and focus on different cell types and their intracellular signaling pathways that could contribute to these processes. We will also discuss the current findings of therapeutic approaches aimed at inhibiting PVR, and preretinal and subretinal fibrosis in both preclinical and clinical investigations. We will also highlight areas that will benefit from additional investigations.

##  METHODS

A comprehensive search was performed on PubMed, Scopus, and ISI databases using each of the following keywords: “subretinal fibrosis”, “proliferative vitreoretinopathy”, “retinal fibrosis”, “fibrovascular membrane”, “ocular fibrosis pathway”, and “therapeutic approaches” in different combinations. All *in vitro* investigations, *in vivo* and preclinical studies, and clinical trials in English were included. No limitation for the time of publication was applied. Only non-English articles, case reports, and abstracts were excluded. In the present study, different posterior segment fibrosis pathways, cell types that may take part in fibrosis, and different treatment modalities were evaluated.

##  RESULTS

### Cell Types Involved in Retinal Fibrosis

Several cell types are considered to be associated with subretinal and preretinal fibrosis. Amarnani et al isolated PVR tissues and cultured their cellular constituents. They showed that PVR membranes were comprised of retinal pigment epithelial (RPE) cells, glial fibrillary acidic protein (GFAP) positive glial cells, and identified cells expressing α -smooth muscle actin ( α SMA), bestrophin-1, and F4/80, suggesting interactions among multiple cell types in PVR.^[8]^ However, the key contribution of these different cell types and their hierarchy to various aspects of fibrosis remains largely unknown.

RPE cells play a key role in visual function, whose dysfunction is associated with various retinal diseases including DR and vitreoretinopathy, age-related macular degeneration (AMD), and retinal detachment.^[9]^ RPE cells also play an important role in retinal fibrosis through their EMT. Increased levels of growth factors and inflammatory cytokines induce RPE cell's EMT, migration, proliferation, and myofibroblast activity. These changes ultimately lead to the formation of membranes and fibrotic tissues.^[10,11]^

In PDR and AMD, Müller glia and astrocytes are activated and produce various ECM proteins and participate in retinal fibrosis. Under disease conditions, Müller cells express α SMA suggesting that they have the potential to transdifferentiate into myofibroblasts.^[12]^ Alternatively, endothelial-to-mesenchymal transition (EndMT) could be contributing to fibrosis. In nAMD the endothelial cells of new blood vessels are activated and may differentiate into myofibroblasts through EndMT contributing to macular fibrosis.^[13]^ In addition, recent studies suggest a critical role for a subset of perivascular supporting cells such as pericytes (PDGFR β ${}^{+}$ Gli1 ${}^{+}$ ) in the repair of tissue damage-mediated fibrosis in many organs, including the eye.^[14]^

Both innate and adaptive immune systems also contribute to subretinal fibrosis. Following a persistent and chronic inflammation, the complement system and various inﬁltrating immune cells including macrophages, neutrophils, and T cells migrate to the retina. Innate immune cells such as neutrophils and monocytes are activated in the initial phase, and as the disease progresses to chronic stages, educated T and B cells may migrate to the damaged retina and participate in repair processes.^[15]^ Macrophages are recruited to the retina under inflammatory conditions and based on* in vitro* cell culture studies, TGF β can induce expression of α SMA, collagen I, and fibronectin in macrophages. Thus, suggesting that macrophages could also undergo macrophage-to-myofibroblast transition (MMT) and contribute to retinal fibrosis. It is estimated that 20% of the cells in the experimental choroidal neovascularization (CNV) are macrophages.^[16,17]^ Circulating fibrocytes and choroidal stromal cells (pericytes and fibroblasts) are the other sources of myofibroblast precursors. A study recently showed that the choroidal pericytes infiltrate the CNV area and differentiate into collagen I-expressing cells.^[18]^ Thus, many ocular cell types get engaged in tissue reparative processes and fibrosis. However, the underlying molecular and cellular mechanisms involved in disease and tissue-specific cellular responses remain unknown.

### Intracellular Signaling Pathways and Posterior Pole Fibrosis

#### TGFβ-signaling pathways

The TGF β and its downstream signaling pathways are most extensively studied during fibrosis in many tissues and organs. The nature of fibrosis in the eye is similar to the fibrotic change in other tissues. Fibrosis results in severe damage to the retinal tissue and cellular functions leading to vision impairment. The exact mechanisms leading to subretinal and preretinal fibrosis remain largely unresolved. However, some known intracellular signaling pathways play important roles in posterior pole fibrosis. In the eye posterior segment, the fibrogenic processes are mainly driven by EMT, which is strictly regulated by the growth factor TGF β . TGF β plays an important role in angiogenesis, ECM production, and tissue repair, as well as cell proliferation and differentiation. TGF β isoforms β 1, β 2, and β 3 have distinct roles in tissue repair. TGF β 1/2 are mainly pro-scarring factors, while TGF β 3 is known for its anti-scarring effect. All isoforms of the TGF β superfamily use the small mothers against the decapentaplegic signaling pathway (Smad) for mediating the downstream signaling events.^[19,20]^

The Smad family is identified as three different subclasses; receptor-activated Smads (R-Smads), common mediator Smads (Co-Smads), and the inhibitory Smads (I-Smads). Smad2 and Smad3 are R-Smads and are phosphorylated upon TGF β binding to its receptor type I kinase. Smad3 is important for the expression of the ECM components, whereas the expression of matrix metalloproteinases (MMPs) is Smad2-dependent.^[21]^ There are several interactions between Smad and non-Smad signaling pathways. For instance, in ARPE-19 cells, TGF β 2 and phosphatidylinositol-3-kinase (PI3K)/Akt pathway were identified to mediate the expression of type I collagen through Smad-dependent and Smad-independent pathways.^[22]^ These findings also suggest a crosstalk between PI3K/Akt, the Smad, and the non-Smad mitogen-activated protein kinases (MAPK), and RhoA/Rho-kinase pathways in ocular fibrotic disorders.^[23]^ In diabetic patients, the PI3K/Akt signaling pathway is activated by high glucose levels in different cell types, such as endothelial cells and podocytes. Akt2 signaling in RPE cells contributes to the RPE EMT in DR. In addition, Akt2 knockout diabetic mice exhibit significantly lower levels of fibrotic changes indicating diabetes-induced retinal ﬁbrosis could be mediated by the PI3K/Akt2/ERK signaling axis.^[24]^

Other molecules or cytokines also affect the TGF β /Smad signaling pathway, such as TNF α that interferes with the activities of TGF β /Smad in the reparative processes.^[25]^ Moreover, CTGF is activated by the TGF β /Smad pathway and enhances fibrosis. CTGF activity is also associated with the process of EMT and ECM synthesis by human ARPE19 cells *in vitro*.^[26]^ CTGF is present at high levels in human PVR membranes and plays a crucial role in the pathogenesis and development of fibrotic retinal diseases. The generation of CTGF is regulated by Yes-associated protein (YAP). YAP is recognized as a vital regulator of EMT in PVR and an important regulator of profibrotic responses in diabetes-induced retinal fibrosis. Furthermore, targeting RhoA/YAP signaling pathways decreases retinal fibrosis.^[27,28,29]^ Platelet-activating factor/platelet-activating factor receptor (PAF/PAF-R) signaling pathway also plays an important role in different fibrotic processes. PAF signaling induces fibronectin expression. RPE cells express PAF-R and are an important source of TGF β . Zhang et al found that PAF-R blockade reduces subretinal fibrosis in a mouse model.^[30]^

#### PDGF/PDGFR signaling pathways

The PDGF-mediated signaling through PDGFR is vital to the proliferation, migration, and survival of stromal cells including perivascular supporting cells and fibroblasts. The PDGF level is increased in the mouse laser CNV model. In this model, the pericytes expressing PDGF-R β migrate early into the subretinal space and contribute to subretinal fibrosis.^[31]^ Blockade of PDGF-R β significantly prevents the recruitment of pericytes to the CNV lesions. In addition, intravitreal injection of anti-PDGF neutralizing antibody could suppress CNV formation and subretinal fibrosis in a preclinical model.^[32]^

#### EGF/EGFR signaling pathway

Epidermal growth factor receptor (EGFR), a member of the ErbB family of receptor tyrosine kinases, plays an important role in the migration and proliferation of epithelial cells including RPE cells. Additionally, epidermal growth factor (EGF) can increase RPE cell survival enhancing their migration and proliferation by activating the EGFR signaling pathway.^[33]^ Activation of EGFR and YAP plays a crucial role in PVR formation. Activated EGFR signaling bypasses RhoA to increase YAP, c-Myc, CyclinD1, and Bcl-xl protein levels.^[34]^

#### MAPK signaling pathways

MAPK signaling pathways could mediate the noncanonical (Smad-independent) TGF β signaling pathways. MAPKs include three subfamilies known as the extracellular signal-regulated kinases (ERKs), p38 mitogen-activated protein kinases (p38s), and the c-Jun N-terminal kinases (JNKs). The ERKs are activated by growth factors and mediate the TGF β 1-induced EMT and fibrosis in ARPE-19 cells.^[35]^ Moreover, the p38 MAPK pathway mediates the expression of type I collagen induced by TGF β 2 in ARPE-19 cells.^[36]^ Myocardin-related transcription factor-A (MRTF-A) activation also contributes to TGF- β -induced EMT in RPE cells. Inhibition of this transcription factor attenuates subretinal fibrosis in a mouse model.^[37]^ Sustained activation of MAPK/ERKs in endothelial cells promotes their EndMT.^[38]^ The RhoA/Rho-kinase pathway is another SMAD-independent pathway, which mediates TGF β action in ocular fibrosis. The RhoA/Rho-kinase pathway mediates the expression of type I collagen induced by TGF β 2 in human ARPE-19 cells.^[39]^ In addition, inhibition of Rho-kinase protects retinal pericytes from high glucose adverse effects.^[40]^

#### Apelin-mediated signaling pathway

Apelin is a bioactive peptide that connects to the angiotensin receptor AT-1-associated receptor protein (APJ). In DR, apelin facilitates the Müller cells' fibrogenic activity through activation of Janus tyrosine kinase 2/Signal transducers and activators of transcription 3 (JAK2/STAT3) signaling pathway. Li et al showed that knockdown of Apelin efficiently inhibited the progression of retinal fibrosis in diabetic rats and decreased the GAFP, collagen I, and α -SMA levels *in vivo* and *in vitro*.^[41]^

#### Toll-like receptor signaling pathways

Toll-like receptors (TLRs) are groups of proteins that play a key role in the innate immune system and innate immune responses contributing to physiological and pathological repair processes including inflammation and fibrosis. Heat shock proteins (HSPs) are a family of highly conserved proteins found in the cytosol and the nucleus of various cell types. HSP70 specifically binds both TLR2 and TLR4.^[42]^ Intraocular administration of HSP70 inhibited subretinal fibrosis in wild-type mice through increased production of IL-10. TLR2- and TLR4-deficient mice showed significant enlargement of the subretinal fibrotic area compared to wild-type mice and failed to upregulate IL-10 expression in response to HSP70.^[43]^ Thus, modulation of IL-10 level contributes to subretinal fibrosis.

#### Other mediators of fibrosis

Sphingolipids are essential for normal physiology and play a role in several pathologies in the retina. Sphingosine-1-phosphate (S1P) and ceramide-1-phosphate (C1P) participate in the proliferation and migration of different cell types.^[44,45]^ S1P and C1P are novel mediators of Müller glia migration, through activation of multiple signaling pathways. Following retinal injuries, the level of S1P and C1P increases resulting in the activation of human RPE cells and their enhanced proliferation, pro-fibrotic and inflammatory responses.^[46,47]^ Both S1P and C1P promote the transcription of pro-inflammatory cytokines interleukin (IL)-6 (IL-6) and IL-8, and EMT marker α -SMA in ARPE-19 cells.^[47]^ Mitigation of signaling through S1P receptor by FTY720 prevents CNV, and likely associated fibrosis, in the mouse laser model of nAMD.^[48]^ The level of periostin (POSTN), a member of the fasciclin (fas) family, is increased in vitreous and fibrovascular membranes from patients with PDR and PVR.^[49,50]^ *In vitro*, periostin increased proliferation, adhesion, and collagen production in RPE cells via focal adhesion kinase (FAK) and AKT phosphorylation.^[50]^ Administration of antisense oligonucleotide directed against periostin inhibited TGF β -induced α SMA expression by 50%.^[48]^

IL-6 secreted by macrophages is a pleiotropic cytokine that plays a role in biological processes such as immune response, inflammation, wound healing, and angiogenesis. A previous study reported that IL-6 induces VEGF and CCL2 expression in vascular endothelial cells leading to the development of CNV.^[51]^ Additionally, in human AMD, high levels of IL-6 in the blood samples from patients with nAMD correlated with disease progression.^[52]^ TGF β 2 promotes IL-6 production in RPE cells.^[53]^ Sato et al found that IL-6 plays a pivotal role in the development of subretinal fibrosis.^[54]^ Blockade of IL-6 receptor significantly decreased subretinal fibrosis and the JAK1–ERK pathway could be involved in the regulation of subretinal scar formation.^[54]^

Caveolin-1 is a membrane protein that plays an important biological role in EMT and CNV, and the growth and migration of microglia/macrophages via JNK activation.^[55]^ Additionally, caveolin-1 plays an important role in the pathogenesis of PVR. Caveolin-1 knock-down and knock-out promoted EMT in both human and mouse RPE cells, whereas increased expression of caveolin-1 blocked EMT.^[56]^ Increased levels of caveolin-1 significantly blocked the RPE cells' EMT, resulting in the reduction of subretinal fibrosis in neovascular AMD. Caveolin-1, however, promotes RPE cellular senescence and the progression of GA in AMD.^[57]^

Wang et al found that accumulation of 7-ketocholesterol (7KC) in drusen increases fibrosis via IQ motif containing GTPase activating protein (IQGAP1). 7KC-induced p21, VEGF, and IL-1 expression were suppressed by an inhibitor of protein kinase C which regulates IQGAP1 serine phosphorylation. This study also demonstrated that mice with a point mutation in IQGAP1 exposed to 7KC show less fibrosis compared to a control group.^[58]^

The α B-crystallin is a member of the small heat-shock protein family and a major protein in the lens and other ocular tissues. The α B-crystallin can induce EMT and enhance TGF β -induced EMT in RPE cells through the Smad4 pathway participating in retinal fibrosis.^[11]^ Galectins are S-type soluble lectins that contribute to several intracellular and intercellular actions. Galectin-1 was found to be upregulated in RPE cells after CNV induction with laser and is associated with subretinal fibrosis through EMT.^[59]^

**Figure 1:**
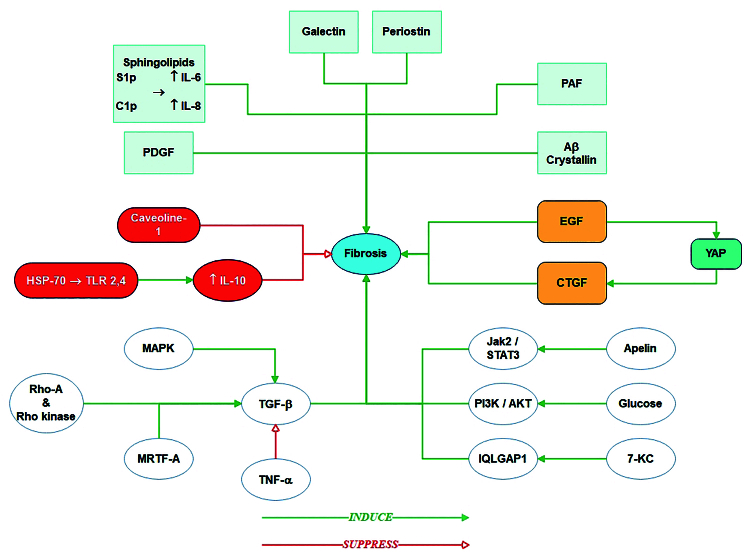
Schematic representation of the molecular mechanisms underlying vitreoretinal fibrosis signaling pathways. Green lines show the induction effects and red lines represent the suppression effects. PAF, platelet-activating factor; EGF, epidermal growth factor; CTGF, connective tissue growth factor; YAP, yes-associated protein; TGF, transforming growth factor; TNF, tumor necrosis factor; MRTF, myocardin-related transcription factor; MAPK, mitogen-activated protein kinase; HSP70, heat shock protein 70; TLR, toll-like receptor; PDGF, platelet-derived growth factor; 7-KC, 7-Ketocholestrol; IQLGAP1, IQ motif containing GTPase activating protein 1; JAK2, Janus kinase 2; STAT3, signal transducer and activator of transcription 3; PI3K, phosphatidylinositol 3-kinase; AKT, protein kinase B.

In summary, much is known about various cellular and biochemical pathways whose dysregulation contributes to the pathophysiology of damage-induced fibrosis [Figure 1]. However, how these cellular and biochemical activities are initiated in various tissues and cells, and how their outcomes are integrated to drive fibrosis remains elusive. Thus, the development of systems biology approaches that allow holistic evaluations of these interactions in a context and tissue and cell-specific dependent manner are vital for the development of effective treatments for mitigating fibrosis.

### Therapeutic Options for Vitroretinal Fibrosis 

#### Preclinical studies targeting pro-fibrosis signaling pathways

Inhibition of TGF β /Smad signaling pathway as the main mediator of ocular fibrosis is effective for the prevention and treatment of PVR. Specifically, the *SMAD7* gene transfer inhibited fibrogenic responses to TGF β 2 in RPE cells *in vitro* and *in vivo*. Expression of the *SMAD7* gene in human RPE cells inhibited the TGF β 2/Smad signaling and expression of type I collagen and TGF β 1. In addition, overexpression of *SMAD7* suppressed EMT and fibrogenic processes in RPE cells after retinal detachment in mice.^[60]^ Alternatively, disruption of the cross-talk between the MAPK pathway and Smad-dependent TGF β signaling pathway could mitigate TGF β profibrotic signaling. The adenoviral gene transfer of dominant-negative p38 MAPK in RPE cells suppressed the postretinal detachment fibrotic responses in a preclinical model of PVR.^[61]^

Fenofibrate, a specific peroxisome proliferator-activated receptor alpha (PPAR α ) agonist, is used clinically to control blood lipid levels. It inhibits Wnt and TGF β – Smad2/3 signaling pathways and suppresses their downstream target CTGF in very low-density lipoprotein receptor knockout (*Vldlr* ${}^{-/-}$ ) mice, a preclinical model of subretinal fibrosis and nAMD. Müller cells are the main source of CTGF; their activation can be blocked by fenofibrate. Protein levels of vimentin, α SMA, collagen I, and fibronectin were significantly decreased in the retina of *Vldlr* ${}^{-/-}$ mice treated with oral administration of fenofibrate. Treatment with Fenofibrate reversed the upregulation of TGF β 2, TGF β -R2, p-Smad2/3, and t-Smad2/3 in retinas from* Vldlr* ${}^{-/-}$ mice, suggesting inhibition of TGF β – Smad2/3 signaling by fenofibrate. Additionally, the antifibrotic effect of fenofibrate, even in the late stage of nAMD, on subretinal fibrosis was noted.^[62]^

Retinoic acid, a metabolite of vitamin A, suppresses TGF β signaling. The retinoic acid receptor α (RAR α ) agonist, Am580, could inhibit collagen gel contraction induced by TGF β 2, suppress the release of IL-6 and expression of EMT markers such as fibronectin, α SMA, and collagen I. It also inhibited the production of pro-MMP2, TIMP-1, and paxillin in RPE cells, and mitigated SMAD2 phosphorylation and MRTF-A nuclear translocation. Thus, Am580 inhibits TGF β 2 effects on RPE cells EMT *in vitro*. Moreover, intravitreal injection of Am580 inhibited subretinal fibrosis in the mouse laser CNV model.^[63]^

Y-27632, a specific inhibitor of the RhoA/ROCK pathway, was investigated to prevent fibrosis in human RPE cells. Y-27632 suppressed the expression of ECM components induced by CTGF or TGF β in ARPE-19 cells *in vitro*.^[64]^ Additionally, several pharmacological inhibitors of the PI3K/Akt signaling pathway have demonstrated, both *in vitro* and *in vivo*, the ability to prevent TGF β -mediated scar formation in eye disorders. For instance, 3-methyladenine, a selective inhibitor of PI3K, exerted antifibrotic effects on experimental subretinal fibrosis in mice.^[65]^ The SB202190, a p38MAPK inhibitor, reduced TGF β 2-mediated migration, and ECM production in the ARPE-19 cells.^[61]^

Another important signaling pathway associated with fibrosis is the Notch signaling pathway, which impacts ocular fibrosis through TGF β 1/Smad axis. Thus, inhibition of this pathway may prevent retinal fibrosis. *In vitro* and *in vivo* experiments have demonstrated that inhibition of the Notch signaling by the γ -secretase inhibitors, including RO4929097, LY411575, and DAPT, could prevent retinal fibrosis.^[66,67,68]^ Intravitreal injection of RO4929097 inhibited retinal glial (Müller) cell-induced gliosis and limited overexpression of ECM proteins in a murine model of retinal fibrosis.^[66]^ LY411575 significantly reduced RPE cell EMT *in vitro* and inhibited PVR formation *in vivo*.^[68]^ Inhibition of the Notch signaling pathway with DAPT suppressed TGF β 2-induced EMT in human RPE cells.^[67]^

Fan et al found that Notch ligands and TGF β 1 had additive effects on the overexpression of ECM proteins in Müller cells. Müller cells treated with Notch ligands upregulated γ -secretase proteases and Notch downstream effectors contributing to fibrosis with increased expression of endogenous TGF β 1, TGF β receptors, and p-Smad3. In addition, an intraperitoneal injection of NaIO ${}_{3}$ in mice caused retinal fibrosis and activated the Notch and TGF β signaling pathways. Intravitreal injection of RO4929097, a selective γ -secretase inhibitor, blocked both Müller cells gliosis and the Notch and TGF β signaling pathways in these mice reducing ECM protein production and suppressing retinal fibrosis. They also found no safety concerns with the intravitreal administration of RO4929097.^[65]^

Bevacizumab as an anti-VEGF agent decreased the expression of VEGFA and VEGFR-1 but did not alter the expression of TIMP1, TIMP2, or α SMA. The level of CTGF expression was elevated after commonly used doses of bevacizumab; however, CTGF was suppressed at a higher dose of anti-VEGF antibody.^[69]^ Treatment with anti-VEGF and anti-CTGF antibodies in combination therapy dramatically inhibited the fibrosis process in mice. Decreased levels of CTGF, MMP2, and TIMP1 expression were shown secondary to injection of anti-CTGF alone or in combination with anti-VEGF.^[69]^ Daftarian et al showed that intravitreal injection of anti-CTGF antibody along with anti-VEGF antibody reduced both neovascular and fibrotic components of CNV membranes in preclinical studies.^[70]^ In an animal model of PVR, intravitreal injection of anti-CTGF antibody alone or in combination with anti-VEGF antibody significantly reduced the mean area of type I collagen fibers of the proliferative membrane.^[71]^

Fibroblast growth factor (FGF) has both angiogenic and fibrotic effects in different diseases. FGF2 induces the growth of vascular endothelial cells, tubular structure formation, and VEGF production.^[71]^ FGF2 could stimulate TGF β 2-induced EMT in RPE cells. FGF2 had a synergetic effect, and the combination of TGF β 2 and FGF2 could cause more EMT than TGF β 2 alone. RBM-007, an aptamer of anti-FGF2, as a new target for the treatment of neovascular AMD in preclinical models, had an inhibitory effect on FGF2-induced angiogenesis, CNV, and subretinal fibrosis. Additionally, RMB-007 had low systemic exposure and a higher half-life in vitreous humor than other available anti-VEGF drugs. Combined intravitreal injection of ranibizumab and RBM-007 had a synergistic inhibitory effect on CNV and subretinal fibrosis.^[73]^

As previously discussed, EGFR signaling plays a critical role in PVR formation via activation of the YAP pathway and increases CTGF as a result. Administration of the EGFR signaling inhibitor, erlotinib, or treatment with the YAP signaling inhibitor, verteporfin, greatly decreased cell cycle progression through downregulation of cyclin D, c-Myc, and Bcl-xl expression. Targeting EGFR and YAP signaling significantly decreased PVR in preclinical models.^[33]^

The antifibrotic and antiangiogenic effects of palmitoylethanolamide (PEA), an endocannabinoid mimetic amide, on neovascular AMD and fibrotic process were evaluated in two animal models, a mouse model of oxygen-induced ischemic retinopathy (OIR) and *Vldlr* ${}^{-/-}$ mice. To investigate angiogenesis effects, PEA was injected intraperitoneally. However, to investigate fibrotic changes PEA was administered orally. It was found that in addition to TGF β /Smad2/3 signaling, α -SMA and fibronectin were increased in OIR and *Vldlr* ${}^{-/-}$ models. PEA could also inhibit retinal neovascularization and had antifibrotic effects in these models via suppression of Müller cell activation and reduced inflammation. These effects were modulated through the activation of the PPAR α pathway and increased levels of the PPAR α protein in the PEA-treated OIR retina.^[74]^

In agreement with other studies, Ma et al found that *Vldlr* ${}^{-/-}$ mice over-activated the TGF β /Smad pathway and induced fibrosis. As a result, overexpression of sVLDLR prevented laser-induced subretinal fibrosis. The antifibrotic effect of sVLDLR was through the inhibition of the canonical Wnt signaling pathway.^[75]^ Plasminogen kringle 5 is a natural angiogenic inhibitor. A rat model of laser-induced epiretinal membrane (ERM) was used to investigate if an intravitreal injection of nanoparticle-mediated delivery of plasminogen kringle 5 (K5-NPs) had inhibitory effects on fibrosis pathways. In this study, K5-NPs effectively inhibited the laser-induced ERM formation and also decreased the expression of fibrosis-inducing cytokines such as TGF β , α SMA, and CTGF.^[76]^ Platelet-activating factor receptor (PAF-R) signaling may be involved in the pathogenesis of subretinal fibrosis. Intravitreal injection of WEB2086, which induced PAF-R blockage in a mouse model of subretinal fibrosis significantly reduced fibrosis.^[30]^

A cyclooxygenase 2 (COX-2)-selective antagonist, NS-398, significantly attenuated subretinal fibrosis through downregulation of TGF- β .^[77]^ COX is a bifunctional rate-limiting enzyme involved in inflammatory immune responses. COX-2 is present in RPE cells, and COX-2 null mice exhibit significantly less CNV formation associated with reduced expression of VEGF.^[78]^ Zhang et al found that COX-2-selective inhibitor reduced subretinal fibrosis *in vivo* and *in vitro*. They also showed that NS-398 could inhibit macrophage accumulation and decrease a proangiogenic state.^[77]^

Many of the growth factors associated with fibrosis mediate their signal through receptor tyrosine kinases, and their inhibition may provide protection against fibrosis. The impact of Nintedanib, a tyrosine kinase inhibitor targeting several receptor tyrosine kinases, on TGF β 2-induced EMT in ARPE-19 cells was evaluated. This investigation demonstrated that Nintedanib treatment could effectively suppress TGF β 2-mediated changes in ARPE-19 cells' E-cadherin expression and ameliorate their enhanced proliferation, migration, and contraction ability.^[79]^

#### Other pathways targeted in preclinical studies

Resveratrol is a polyphenol phytoalexin found in red wine with anti-oxidative, anti-inflammatory, and anti-proliferative properties. Chan et al showed that resveratrol inhibits fibrosis by inhibiting PDGFBB-induced migration and signaling in ARPE-19 cells via PDGFR β , PI3K/Akt, and MAPK pathways.^[80]^ In addition, resveratrol effectively inhibited cell migration and EMT by suppressing phosphorylation of Smad2 and Smad3 in TGF β 2-treated RPE cells.^[81]^ Resveratrol also inhibited the progression of experimental PVR in a preclinical study.^[82]^ Dual treatment of human RPE cells in culture with bevacizumab and resveratrol reduced EMT more effectively than bevacizumab alone. This study showed that resveratrol reversed the adverse effects of bevacizumab via the Notch signaling pathway.^[83]^ These results suggest the use of resveratrol as a therapeutic agent for the prevention of PVR progression.

Simon et al found that S1P and C1P addition to the RPE cells (ARPE-19 and D407 cell lines) significantly enhanced their migration, and pretreatment with W146 and BML-241, S1P receptors antagonists, blocked exogenous S1P-induced cell migration. Inhibiting sphingosine kinase 1 (SphK1), the enzyme involved in S1P synthesis, also significantly reduced cell migration. Whereas inhibiting C1P synthesis did not affect C1P-induced migration. These studies suggest an essential role for S1P and C1P in the regulation of RPE cell migration and inflammation.^[47]^ Exosomes secreted from mesenchymal stem cells (MSCs) could elicit significant therapeutic effects by suppressing fibrosis in several retinal injury models.^[84,85]^ Treatment with intravitreal injection of MSC-derived exosomes slowed the growth of CNV and reduced the number of fibroblasts and collagen fibers after laser photocoagulation. Thus, indicating that MSC-derived exosomes have great potential to reduce collagen deposits in subretinal fibrosis.^[84]^ Additionally, intravitreal injection of MSC-derived exosomes could be maintained in the vitreous for more than four weeks and had a prolonged therapeutic effect, requiring fewer injections.^[86]^ Human umbilical cord-derived mesenchymal stem cell exosomes (hucMSC-Exo) could inhibit cell migration and expression of EMT-associated proteins, which contributes to suppressing EMT. The therapeutic effect of hucMSC-Exo was attributed to miR-27b activity, targeting the homeobox protein (Hox-C6) gene, which could directly inhibit EMT in RPE cells. The hucMSC-derived miR-27b could alleviate subretinal fibrosis by inhibiting the EMT process in RPE cells.^[87]^

The renin-angiotensin system (RAS) has a distinct role in angiogenesis and inflammation. (Pro)renin receptor (PRR), encoded by the *ATP6AP2* gene and prorenin binding to (pro)renin receptor, activates the RAS and RAS-independent signaling, and their activation contributes to the molecular pathogenesis of various ocular diseases. The inhibition of PRR by intravitreal injection of PRR proline-modified short hairpin RNA (PRR-P sh-RNA) inhibited CNV formation, CNV-related inflammatory molecule expression, macrophage infiltration, and ERK1/2 activation. It also suppressed both TGF β expression and inflammation-related angiogenesis and fibrosis. It was also noted that the therapeutic effect of PRR-PshRNA was comparable with aflibercept.^[88]^ GEF-H1/ARHGEF2, a guanine nucleotide exchange factor for RhoA (GEF), has a role in inflammatory and fibrotic processes. GEF-H1 regulates the interaction between microtubules and the actin cytoskeleton, a process that is important during cell contractility and migration, cell shape changes, and intercellular junction remodeling, as well as cell proliferation and mitosis.^[89]^ Clare et al generated peptide inhibitors to block the GEF-H1 signaling pathway. The most potent one, TAT P5, could suppress TGF- β and LPS signaling pathways, decrease cell migration, and downregulate junctional proteins in preclinical models of retinal diseases.^[90]^

Periostin (POSTN) which is produced by RPE cells has an important role in the formation of preretinal and choroidal fibrovascular membrane. The expression of POSTN is enhanced in both the retina and choroid after laser treatment in the mouse laser CNV model that picks three days after laser. However, the expression of POSTN is significantly more prominent in the mouse choroid compared to the retina. In addition, the major source of POSTN in the retina is the retinal perivascular cells (pericytes), while the choroid endothelial cells, followed by RPE cells and pericytes, express significantly higher levels of POSTN compared to retinal pericytes (our unpublished data). Using a new class of RNA interference (RNAi) agent (NK0144) targeting POSTN, Nakama et al showed that intravitreal injections of NK0144 significantly inhibited the volume of the induced CNVs and subsequent fibrosis.^[91]^

Doxycycline is a semi-synthetic tetracycline, which inhibits inflammation and cell proliferation via various mechanisms, including MMPs, PI3K/Akt-eNOS pathways, and FasL.^[92]^ Doxycycline suppressed M2 polarization and subsequently attenuated EMT and subretinal fibrosis via the signal transducer and activator of the transcription 6 (STAT6) pathway in RPE cells. It also inhibited pro-fibrotic/angiogenic macrophages and the subsequent angiogenesis and fibrosis processes.^[93]^

Adrenomedullin (AM) is a vasoactive peptide that regulates vascular homeostasis and inhibits fibrosis through interactions with receptor activity modifying protein 2 (RAMP2) reducing oxidative stress. Tanaka et al found that AM and RAMP2 knockout mice exhibit enhanced neovascular formation, subretinal fibrosis, and macrophage invasion compared to wild-type mice. They also showed that an intravitreal injection of AM suppressed fibrosis and significantly reduced fibrosis-related molecules in the mouse laser CNV model. Thus, AM-RAMP2 interactions could suppress EMT and mitigate subretinal fibrosis.^[94]^

Amarnani et al showed that the PVR membrane is comprised of multiple cell types. Additionally, this study demonstrated that methotrexate (MTX), an antimetabolite and antifolate drug, significantly decreased cell proliferation and band formation, but had no significant impact on cell migration. MTX also regulated cell death via activation of the caspase 3/7 pathway.^[8]^ Daunorubicin (DNR) is a potent cell proliferation inhibitor and is effective against PVR. Xiao et al demonstrated that DNR and dexamethasone loaded over silicone particles strongly suppressed cell proliferation in an animal model of PVR. They showed that dual treatment is significantly superior compared to single-drug treatment.^[95]^ They previously showed that DNR-loaded silicone particles were safe and stayed significantly longer in the vitreous cavity and provided two to three months of therapeutic drug levels.^[96]^

The epigenetic modifications, including DNA methylation and histone acetylation, could regulate the EMT of RPE cells. Methyl-CpG-binding protein 2 (MeCP2) is the prototypic methyl-CpG-binding protein, which binds to methylated DNA through a conserved methyl-CpG-binding domain where they typically suppress gene expression and is expressed extensively in cells within PVR membranes. 5-aza-20-deoxycytidine (5-AZA-dC) is a potent inhibitor of DNA methylation. He et al demonstrated that treatment with 5-AZA-dC significantly suppressed the expression of α SMA, TGF β -R2, and phosphorylation of Smad2/3, and inhibited RPE cell migration. TGF β -induced expression of α SMA was suppressed by the knockdown of MeCP2.^[97]^

Histone deacetylases (HDACs)-mediated epigenetic mechanisms play important roles in the regulation of RPE cell proliferation and EMT. Trichostatin A (TSA), a class I and II HDAC inhibitor, inhibited the proliferation of RPE cells by suppressing the G1 phase cell cycle through repression of cyclin/CDK/p-Rb and induction of p21 and p27. TSA strongly mitigated TGF β 2-induced morphological changes and the upregulation of α SMA, type I and type IV collagens, and fibronectin. Thus, the inhibition of HDAC activity with TSA strongly attenuated the proliferation and TGF β 2-induced EMT in human RPE cells.^[98]^

Epigenetic reprogramming in EMT is an interesting approach to fibrosis as it provides a long-term, stable, and reversible regulation. N6-methyladenosine (m6A) is the most common epigenetic modification of mRNA, which mediates more than 80% of RNA methylation. Ma et al evaluated the impact of m6A methyltransferase METTL3 expression on ARPE-19 cells EMT. Additionally, in this study, intravitreal injection of cells overexpressing METTL3 was used to assess the impact of METTL3 on the establishment of the Wnt/ β -catenin pathway in ARPE-19 cells and eventually suppress the expression of several proteins belonging to the Wnt/ β -catenin pathway. *In vivo* investigations showed that METTL3 could delay the initiation and development of PVR in a rat model.^[99]^ Wang et al found that METTL3 was upregulated in RPE cells during subretinal ﬁbrosis in the mouse laser CNV model. METTL3-mediated m6A modiﬁcation has a critical role in RPE cell EMT. METTL3 deﬁciency in RPE cells resulting from adeno-associated virus (AAV) knockdown significantly attenuated subretinal fibrosis in this model of AMD [Table 1].^[100]^

Luteolin is a type of flavonoid, whose intravitreal administration in a laser-induced mouse model of CNV showed antifibrotic activity. Luteolin inhibited EMT in RPE cells through inactivation of Smad2/3 and YAP signaling. In addition, an *in vitro* study revealed that luteolin significantly inhibits fibronectin, α -SMA, N-cadherin, and vimentin expression.^[101]^ Pirfenidone (PFD) is a new type of broad-spectrum antifibrotic complex that exerts antioxidant, anti-inflammatory, and antifibrotic activity. PFD can inhibit CTGF, PDGF, α SMA, and TGF β expression, and consequently delay or even reverse fibrosis and scar formation. In the mouse laser CNV model, PFD suppressed the TGF β /Smad signaling pathway and attenuated α SMA and collagen I expression.^[102,103]^

### Therapeutic Options for Vitroretinal Fibrosis

#### Clinical studies

Several clinical studies have investigated the therapeutic potential of various agents for the prevention and treatment of posterior segment fibrosis. The possible effect of intravitreal daunorubicin on traumatic PVR was assessed in the late 80s.^[104]^ Subsequently, a multicenter RCT revealed that daunorubicin could not significantly increase the treatment success rate in eyes with RRD and PVR; however, a tendency toward reduced rate of reoperations was observed [Table 2].^[105]^

The VEGF concentration is elevated in the vitreous of patients with PDR and PVR grade C.^[106]^ Ghasemi Falavarjani et al determined whether intra-silicone oil injection of bevacizumab could eliminate PVR formation in patients undergoing vitrectomy for RRD and PVR grade C. They noticed no differences between the groups that received intra-silicone bevacizumab and those without bevacizumab injection.^[107]^ Tousi et al also evaluated the effect of intravitreal bevacizumab (IVB) as a surgical adjunct in the prevention of PVR after RD surgery.^[108]^ The preliminary results showed neither a benefit nor any harm from this intervention in terms of the anatomic and visual outcomes.

Jonas and coworkers showed that intravitreal crystalline cortisone was well tolerated by eyes undergoing pars plana vitrectomy for PDR complications.^[109]^ Although studies showed intravitreal or subtenon injection of triamcinolone acetonide to be safe and that the adverse effects were not higher than the normal rate, they could not identify its beneficial impact on PVR.^[110,111,112,113]^ In patients who underwent vitrectomy with silicone oil for PVR, a slow-release dexamethasone implant did not improve the primary anatomic success rate but significantly improved the cystoid macular edema.^[114]^ Additionally, another study demonstrated that intravitreal injection of triamcinolone in silicone-filled eye did not significantly impact PVR processes.^[115]^ Dehghan et al showed that oral prednisolone prescribed after scleral buckling in simple phakic RRD did not significantly improve the anatomic and visual outcomes.^[116]^

Colchicine was suggested to inhibit cell proliferation by binding tubulin and inhibiting microtubule proliferation.^[117]^ However, a placebo-controlled randomized clinical trial revealed that oral colchicine did not have a significant effect on reducing the rate of retinal re-detachment due to PVR in eyes undergoing scleral buckling.^[118]^

**Table 1:** Summary of preclinical studies for treating the experimental posterior segment fibrosis

**Reference**	**Year**	**Therapy**	**Model**	**Route of administration**
Saika et al^[61]^	2005	Inhibition of p38MAPK	Mouse PVR model	Intravitreal/gene transfer
Saika et al^[60]^	2007	*SMAD7* gene overexpression	Mouse PVR model	Intravitreal
Zhu et al^[64]^	2013	Inhibition of RhoA/Rho-kinase pathway	*In vitro* ARPE-19 cells	–
Zhang et al^[30]^	2013	Platelet-activating factor receptor antagonist	Mouse laser-induced CNV	Intraperitoneal
Chen et al^[67]^	2014	Inhibition of Jagged/Notch pathway	*In vitro* human RPE cells	–
Xiao et al^[98]^	2014	Trichostatin A	*In vitro* RPE cells	–
Ishikawa et al^[82]^	2015	Resveratrol	*In vitro* human RPE cells AND Rabbit PVR model	Intravitreal
Nakama et al^[91]^	2015	Periostin antagonism	Mouse laser-induced CNV	Intravitreal
He et al^[97]^	2015	Inhibition of DNA methylation and methyl-CpG-binding protein 2 (MeCP2)	*In vitro* patient-derived model of PVR	–
Zhang et al^[77]^	2016	COX-2-selective antagonist (NS-398)	Mouse laser-induced CNV	Intravitreal
Subramani et al^[83]^	2017	Resveratrol	*In vitro* ARPE-19 cells	–
Zhang et al^[68]^	2017	Inhibiting the Notch signaling activation	Mouse PVR model	Intravitreal
Lu et al^[76]^	2017	Nanoparticle-mediated delivery of plasminogen kringle 5 (K5-NPs)	Rat laser CNV	Intravitreal
Amarnani et al^[8]^	2017	Methotrexate	*In vitro* patient-derived model of PVR	–
He et al^[84]^	2018	Mesenchymal stem cells-derived exosomes	Mouse laser-induced CNV	Intravitreal
Peng et al^[93]^	2018	Doxycycline	Mouse laser-induced CNV	Intraperitoneal
Daftarian et al^[70]^	2019	Connective tissue growth factor neutralizing antibody(anti-CTGF)	Rat laser CNV	Intravitreal
Matsuda et al^[73]^	2019	Anti-fibroblast growth factor 2 aptamer	Mouse and rat laser-induced CNV	Intravitreal
Mathew et al^[86]^	2019	MSC-derived exosomes	Rat retinal ischemia	Intravitreal
Liu et al^[88]^	2019	Novel RNAi therapeutic agent against (Pro)renin receptor	Mouse laser-induced CNV	Intravitreal
Chen et al^[62]^	2020	Fenofibrates	Vldlr (–/–) mice subretinal fibrosis model	Oral administration
Bo et al^[65]^	2020	3-methyladenine, a selective inhibitor of PI3K	Mouse laser-induced CNV	Intravitreal
Fan et al^[66]^	2020	Inhibiting the Notch and TGF- β signaling pathways	Mouse sodium iodate (NaIO ${}_{3}$ )-induced retinal injury	Intravitreal
Ye et al^[74]^	2020	Palmitoylethanolamide (PEA)	Mouse oxygen-induced retinopathy (OIR) + (Vldlr - / - )	Intraperitoneal + orally
Xiao et al^[95]^	2020	Daunorubicin (DNR) and dexamethasone	Rabbit PVR model	Intravitreal
Kobayashi et al^[63]^	2021	Retinoic acid receptor- α agonist (AM540)	Mouse laser-induced CNV	Intravitreal
Gao et al^[103]^	2021	Pirfenidone (PFD)	Mouse laser-induced CNV	Intravitreal
Daftarian et al^[71]^	2021	Connective tissue growth factor neutralizing antibody(anti-CTGF)	Rabbit PVR model	Intravitreal
Li et al^[87]^	2021	Human umbilical cord-derived mesenchymal stem cell exosomes (hucMSC-Exo)	Mouse laser-induced CNV	Intravitreal
Tanaka et al^[94]^	2021	Adrenomedullin	Mouse laser-induced CNV	Intravitreal
Ma et al^[99]^	2021	METTL3 - Wnt/ β ‐catenin pathway	Rat PVR model	Intravitreal
Simón et al^[47]^	2022	Sphingosine-1-phosphate and ceramide-1-phosphate	*In vitro* ARPE-19 cells	–
Zhang & Li^[33]^	2022	Epidermal growth factor receptor (EGFR) and the yes-associated protein (YAP) signaling pathway	Mouse PVR model	Intravitreal
Mills et al^[90]^	2022	Inhibition of GEF-H1 signaling pathway	Mouse laser-induced CNV	Intravitreal
Yin et al^[79]^	2023	Nintedanib	*In vitro* ARPE-19 cells	–
Zhang et al^[101]^	2023	Luteolin	Mouse laser-induced CNV	Intravitreal
Wang et al^[100]^	2023	METTL3	Mouse laser-induced CNV	Intravitreal
CNV, choroidal neovascularization, PVR, proliferative vitreoretinopathy, RPE, retinal pigment epithelium, TGF, transforming growth factor				

**Table 2:** Summary of clinical trial studies with relevant antifibrotic drugs for treating human posterior segment fibrosis

**Author**	**Year**	**Therapy**	**Model**	**Route of administration**
Wiedemann et al^[105]^	1998	Daunorubicin	PVR – 286 patients	Intravitreal
Jonas et al^[109]^	2000	Crystalline cortisone	PDR – 32 eyes	Intravitreal
Asaria et al^[120]^	2001	5-FU	PVR – 174 patients	Intravitreal
Jonas et al^[112]^	2003	Triamcinolone acetonide	PDR – 32 eyes	Intravitreal
Kumar et al^[119]^	2003	LMWH	Advanced PVR – 30 patients	Intravitreal
Charteris et al^[122]^	2004	5-fluorouracil & LMWH	PVR – 157 pateint	Intravitreal
Munir et al^[110]^	2005	Triamcinolone acetonide	PDR – 13 eyes	Intravitreal
Cheema et al^[113]^	2007	Triamcinolone acetonide	PVR – 24 patients	Intravitreal – intra-silicone oil
Garcia et al^[123]^	2007	5-fluorouracil & LMWH + silicone oil	PVR – 33 eyes	Intravitreal
Wickham et al^[124]^	2007	5-fluorouracil & LMWH	RRD, PVR - 641 patient	Intravitreal
Ahmadieh et al^[115]^	2008	Triamcinolone acetonide	PVR –75 eyes	Intravitreal – intra-silicone oil
Dehghan et al^[116]^	2010	Prednisolone	PVR – 52 eyes	Oral
Lee et al^[111]^	2013	Adjunctive subtenon injection of triamcinolone acetonide (TA)	PDR – 27 eyes	Subtenon
Ghasemi Falavarjani et al^[107]^	2014	Bevacizumab	Advanced PDR – 38 eyes	Intravitreal – intra-silicone oil
Ganekal & Dorairaj^[121]^	2014	5-fluorouracil & LMWH	PVR – 40 patients	Intravitreal
Ahmadieh et al^[118]^	2015	Colchicine	PVR – 184 patients	Oral
Ghasemi Falavarjani et al^[127]^	2015	MTX	PDR – 38 eyes	Intravitreal – intra-silicone oil
Tousi et al^[108]^	2016	Bevacizumab	PVR – 27 patients	Intravitreal
Sadaka et al^[126]^	2016	MTX	Severe recurrent PVR – 29 eyes	Intravitreal
Banerjee et al^[114]^	2017	Dexamethasone implant	PDR – 140 patients	Intravitreal
Nourinia et al^[128]^	2019	MTX	PVR – 11 eyes	Intravitreal – intra-silicone oil
PDR, proliferative diabetic retinopathy; PVR, proliferative vitreoretinopathy; MTX, methotrexate; LMVH, low molecular weight heparin; 5-FU, 5-fluorouracil				

Low molecular weight heparin (LMWH) has been shown to reduce postoperative fibrin formation after vitrectomy. LMWH acts by binding to fibronectin which is the most potent stimulator of RPE cell migration, and also inhibits PDGF, EGF, and FGF stimulation of RPE cell migration and proliferation.^[119]^ The other drug used to eliminate PVR is 5-FU, which inhibits DNA synthesis and fibroblast proliferation. Asaria and coworkers demonstrated significant inhibition of postoperative PVR formation in patients with RRD who underwent intraoperative adjuvant combination therapy with LMVH and 5-FU.^[120]^ However, other studies revealed that perioperative infusion of combined 5-FU and LMWH could not prevent postoperative PVR or increase the success rate of surgery in established PVR.^[121,122]^ Garcia et al also demonstrated that intraoperative infusion of 5-FU and LMWH in patients with RRD, and PVR did not impact the retinal reattachment rate and prognosis of visual acuity.^[123]^ Finally, a study reported the worse visual outcome in the group that received an intraoperative infusion of 5-FU and LMWH in patients with macular sparing RRD, and it was recommended that the adjuvant 5-FU and LMWH infusion during vitrectomy should not be used routinely in patients who are at high risk for PVR.^[124]^

Methotrexate (MTX) may reduce PVR formation by suppressing ocular inflammation.^[125]^ In a study by Sadaka et al, intravitreal MTX infusion (IMI) during pars plana vitrectomy was used in retinal detachment repair in patients with high risk for PVR. This study showed no PVR induction in eyes treated with IMI and 90% of retinas remained attached during 27 months of follow-up.^[126]^ Intra-silicone oil injection of MTX was evaluated in patients with tractional macular or retinal detachment due to advanced PDR. However, there were no significant differences between the groups that received intra-silicone oil MTX at the end of surgery and the control group.^[127]^ In a pilot study, repeated intra-silicone oil injections of MTX were assessed in 11 patients with PVR grade C and the retina remained attached for nine months follow-up.^[128]^ Promising results of the pilot studies on the effects of MTX on PVR led to the design of two randomized controlled trials (https://www.clinicaltrials.gov/: NCT04482543 and NCT04136366).

##  DISCUSSION 

Posterior segment fibrosis is a complex biological phenomenon and is responsible for many retinal disorders leading to severe visual impairment. Subretinal fibrosis in nAMD, fibrovascular tissue formation in PDR, and unsuccessful retinal detachment surgery due to PVR, all are examples of the destructive nature of fibrotic pathways in vitreoretinal diseases. Current evidence suggests that preretinal and subretinal fibrosis are formed by contributions from several cell types engaged via multiple pathways. RPE, choroidal stromal cells, pericytes, endothelial cells, glial cells, and immune cells like macrophages contribute to ECM production and fibrosis. However, how the activity of these cells and their unique contributions are coordinated during fibrosis remains an unmet challenge.

Several studies have shown that fibrotic processes progress despite successful anti-VEGF therapy. Citirik et al showed an elevated VEGF level in PDR and in patients with PVR.^[106,129]^ Clinical trials have demonstrated no significant difference in PVR formation between patients who received anti-VEGF during or after vitrectomy and the control group. However, other preclinical studies have shown that concurrent inhibition of VEGF and CTGF has a synergistic effect and significantly inhibits fibrosis.^[69,70]^

We discussed some agents like fenofibrate, retinoic acid receptor α agonists, and pirfenidone that suppressed several fibrosis pathways and significantly inhibited fibrosis in preclinical studies. However, currently, there are no ongoing clinical studies investigating the effectiveness of these agents in suppression of retinal fibrosis. The antifibrotic effects of daunorubicin, MTX, triamcinolone, 5-fluorouracil, and low molecular weight heparin (LMWH) were investigated mainly in PDR and PVR patients in a number of clinical trials. The outcomes of these clinical trials may be a good starting point for additional future investigations.

Similar to fibrosis in other tissues, TGF β and Smad pathways play an essential role in posterior segment fibrosis. Although several signaling pathways result in fibrosis, investigations on new molecules which may have an impact on retinal fibrotic changes are still ongoing. Here we discussed major studied pathways such as TGF β , CTGF, PDGF, EGF, galectin, periostin, caveolin, PAF, HSP-70, sphingolipids, and crystalline proteins, and most recently sonic hedgehog signaling, which interact with posterior segment fibrosis. Inhibition of one signaling pathway may cause overexpression of other pathways. The knowledge of the pathophysiology of multiple signaling pathways in scar formation demonstrates the complexity of the process and the limitation of relevant clinical success by inhibiting a single factor.

Daunorubicin is an anthracycline antibiotic that inhibits DNA synthesis and DNA-dependent RNA synthesis. Previous investigations showed that daunorubicin suppresses the fibrotic process and combination therapy with dexamethasone in an animal model of PVR is significantly more effective than single therapy.^[95]^ Intravitreal injection of daunorubicin at the end of vitrectomy in patients with RRD and PVR, however, did not significantly increase the retinal reattachment rate.^[130]^

MTX inhibits dihydrofolate reductase and interferes with DNA synthesis, repair, and cellular replication. Basic research studies showed that MTX significantly decreased cell proliferation and band formation but had no significant impact on cell migration in a patient-derived culture of PVR tissue.^[8]^ Sadaka et al showed that intravitreal infusion of MTX decreased PVR formation with high attachment rate.^[126]^ However, a single intra-silicone oil injection of MTX in patients with RRD and high risk for PVR was not superior compared to the control group.^[127]^ Further clinical studies using multiple intravitreal or intra-silicone oil injections are on the way.

##  SUMMARY

Several cell types and mediators interact in a coordinated manner to mediate posterior segment fibrosis in the eye. Further investigations are needed to reduce the destructive impact of fibrosis on vitreoretinal diseases. Current treatment modalities are not sufficiently effective to inhibit or prevent the fibrotic processes. Thus, new and alternative treatments need to be evaluated in clinical settings for effective mitigation of ocular fibrosis. In the present review, we introduced several intracellular signaling pathways, which are engaged during fibrosis and discussed the results of *in vitro* and *in vivo* targeting of these pathways. Epigenetic reprogramming also seems effective in the long-term prevention and treatment of fibrotic processes and needs further evaluation. Recently a tyrosine kinase inhibitor, nintedanib, successfully decreased fibrosis *in vitro*. However, it's *in vivo* clinical benefits await further research and clinical investigation. Efforts to expand our knowledge regarding the molecular and cellular pathways involved in fibrosis and their coordinated impact will aid in the development of new and effective treatment modalities.

##  Financial Support and Sponsorship

None.

##  Conflicts of Interest

None.
